# Clinical significance of post-liver transplant hepatitis E seropositivity in high prevalence area of hepatitis E genotype 3: a prospective study

**DOI:** 10.1038/s41598-020-64551-x

**Published:** 2020-04-30

**Authors:** Piyawat Komolmit, Vinita Oranrap, Sirinporn Suksawatamnuay, Kessarin Thanapirom, Supachaya Sriphoosanaphan, Nunthiya Srisoonthorn, Nawarat Posuwan, Thanunrat Thongmee, Sombat Treeprasertsuk, Yong Poovorawan

**Affiliations:** 1 Division of Gastroenterology, Department of Medicine, Faculty of Medicine, Chulalongkorn University, Bangkok, Thailand; 2 The Research Unit of Hepatic Fibrosis and Cirrhosis, Department of Medicine, Chulalongkorn University, Bangkok, Thailand; 3 Center of Excellence in Liver Diseases, King Chulalongkorn Memorial Hospital, Bangkok, Thailand; 4 Center of Excellence in Clinical Virology, Faculty of Medicine, Chulalongkorn University, Bangkok, Thailand

**Keywords:** Hepatology, Viral epidemiology, Hepatitis

## Abstract

High hepatitis E (HEV) seroprevalence has been reported in the general population and in post-liver transplant (LT) cases in several regions, including Thailand, with genotype 3 being a predominant genotype. We hypothesized that HEV might persist at a subclinical level and might pose clinical risks in the post-LT period. We performed a cross-sectional study with 108 post-LT patients and found an IgG seroprevalence of 55.6%. Subsequently, 91 cases without clinical evidence of HEV-related hepatitis were enrolled in 1 year of prospective follow-up to determine clinical status, serologies and serum/feces HEV RNA every 4 months. HEV RNA was detected, indicating subclinical infections in patients with or without seropositivity, with an annual incidence of 7.7%. Our results suggest that subclinical HEV infection exists among LT patients in this high-prevalence area. Thus, clinicians should be aware of the possibility of disease reemergence and HEV viral transmission in LT patients.

## Introduction

It has been a decade since the emergence of evidence of the ability of hepatitis E virus (HEV) to cause chronic hepatitis and cirrhosis in immunocompromised hosts, including HIV and solid organ transplant (SOT) patients, and especially in liver transplant (LT) recipients^1,2^. Of the four genotypes of HEV causing hepatitis in humans, genotypes 1 and 2 are transmitted via the fecal-oral route, causing endemic outbreaks and acute self-limited hepatitis of varying severity, and are common in tropical and non-industrialized countries, such as India and Pakistan^3^. Genotypes 3 and 4 are zoonotic, with pigs, wild boar and other animals serving as their intermediate hosts and are transmitted through uncooked meat^3^.

HEV genotype 3 is the specific genotype causing chronic hepatitis, with a rare report of genotype 4^3,4^. It is commonly found and reported to cause chronic infection in SOT patients in several regions of Europe. Interestingly, Thailand has genotype 3 as a common genotype, unlike other neighboring countries in Southeast Asia, India and China^5^. This gives unique insight into the hidden problems in SOT recipients that have never been explored. The prevalence of positive HEV IgG antibodies in blood donors varies among the areas from 8.9–37.3% in the Thai population, which is quite high compared to 2–16% in some low-prevalence areas in developed countries in Europe^6–9^.

The HEV IgG seroprevalence in SOT and LT recipients has been reported in several studies in Europe and North America, varying from 3–28%, with a serum RNA detection rate of 0–2%, and the incidence of chronic hepatitis E is up to 3%^10^. The positivity of HEV serology in this setting depends on the immunosuppressive status of the patients and the detection ability of the commercial tests^11,12^. Therefore, the diagnosis of the disease relies upon the detection of HEV RNA in the serum or stool^3^.

The significance of HEV infection and the clinical course of the disease in SOT patients are debated. The variations in HEV seroprevalence and the incidence of active disease in different countries lead to different conclusions regarding the clinical significance and give rise to different views of the practical implications^13,14^. The clinical studies of HEV in SOT recipients thus far have addressed the problem of HEV from the perspective of a series of selected case reports, cross-sectional studies and retrospective cohorts^10^. Without a prospective study, it is challenging to determine the extent of the problem of diseases associated with HEV genotype 3 in the SOT population, whether the patients with IgG positivity can clear the virus, whether the patients without IgG seropositivity remain at risk without specific RNA testing, and what are the clinical courses of the patients who are positive for HEV RNA. To clarify some of the above issues, we conducted a clinical study in post-LT patients to determine the seroprevalence. Subsequently, the patients were prospectively followed for 12 months, and the clinical changes, liver function tests, serum HEV serology and RNA, and stool HEV RNA were assessed.

## Results

**Figure 1:**
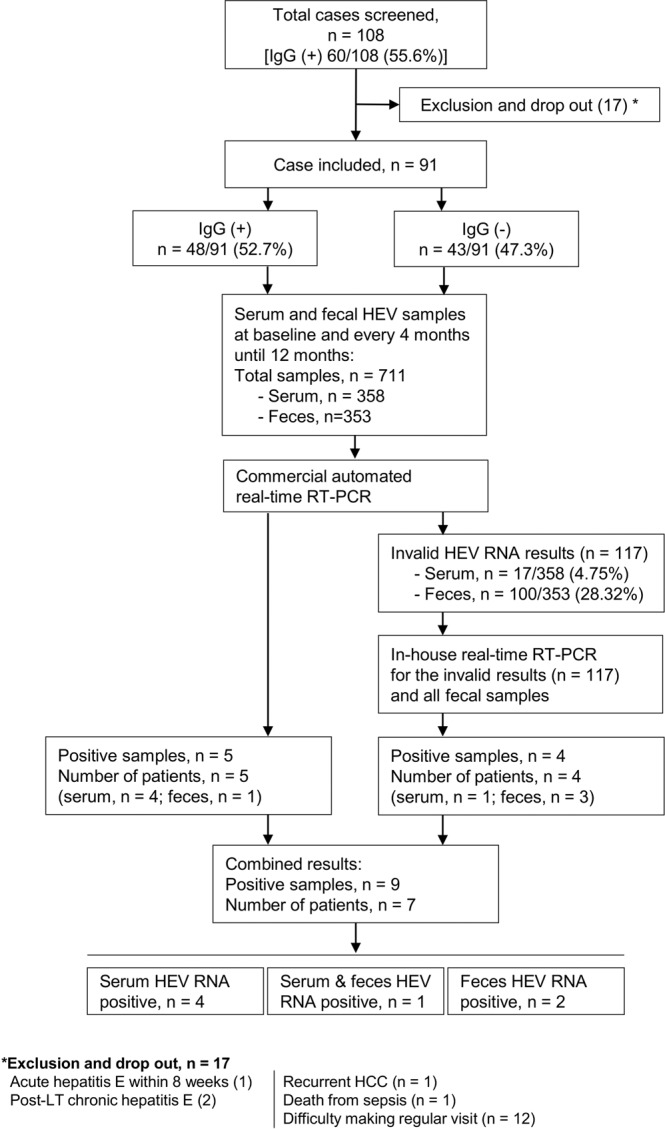
Flow diagram for patient recruitment and hepatitis E RNA assessment. Post-liver transplant patients were screened for serum hepatitis E IgG. After recruitment, all patients were assessed and followed-up every 4 months for 12 months. All serum and fecal samples were collected per the protocol and assessed for HEV RNA at the end of the study using a commercial real-time RT PCR kit, COBAS HEV. If the test result was invalid, the sample was subsequently tested in-house by real-time RT-PCR. The numbers of invalid samples, positive samples and patients in each step are shown.

Of the 108 post-LT patients evaluated for HEV seroprevalence, 60 (55.6%) were seropositive [HEV IgG (+)], and 48 (44.4%) were seronegative [HEV IgG (−)] (Fig. 1). Seventeen patients were excluded before entering the follow-up study. Three patients (2.8%) were diagnosed with HEV-related acute or chronic hepatitis. One patient with chronic hepatitis E, identified as being caused by HEV genotype 3, was on the second month of treatment, and the other had previously been treated with ribavirin. A patient was diagnosed with acute hepatitis E and started the treatment at 10 weeks post-LT. The other 14 patients included a patient who died a few months later from severe sepsis, a patient who had recurrent hepatoma, and 12 who were unable to undergo regular follow-up visits. Ninety-one patients were recruited for the follow-up period. Of these, forty-eight patients (52.7%) were HEV IgG (+).

The baseline characteristics of the HEV IgG (+) and HEV IgG (−) groups are shown in Table 1. The two groups were not different in age, sex or time of recruitment. Thirteen patients (14.3%) had a previous history of ACR requiring steroid pulse therapy before enrollment. There was no significant difference between the groups. The overall prevalence of diabetes in this study was 49.5%. There was no difference in the proportion of patients with diabetes between the groups.

**Table 1:** Baseline characteristics of the patients with anti-HEV IgG seropositivity (+) and anti-HEV IgG seronegativity (−). Data are shown as the mean ± SD.

Variables	Seropositive	Seronegative	p-value
Number of patients, n/total (%)	48/91 (52.7)	43/91 (47.3)	-
Age (years)	57 ± 12	59 ± 13	0.523
Male sex, n/total (%)	33/48 (68.8)	30/43 (69.6)	0.916
Time of the first test after LT* (months), [range]	70 [15–172]	75 [19–201]	0.615
**Indication for LT, n/total (%)**			
Hepatitis B	20/48 (41.7)	14/43 (32.6)	0.394
Hepatitis C	14/48 (29.2)	14/43 (32.6)	0.821
Alcohol	11/48 (22.9)	7/43 (16.3)	0.599
Hepatocellular carcinoma	21/48 (43.8)	22/43 (51.2)	0.532
Others	7/48 (14.6)	10/43 (23.3)	0.420
MELD-Na score* (Q1-Q3)	7 (6.25–9.0)	7 (6.0–9.0)	0.590
**Hepatitis C treatment**			
IFN/RBV, n/total (%)	5/48 (10.4)	8/43 (18.6)	0.208
DAA/RBV, n/total (%)	6/48 (12.5)	4/43 (9.3)	0.442
Diabetes	23/48 (47.9)	22/43 (51.2)	0.757
ACR requiring pulse steroid therapy	7/48 (14.6)	6/43 (14.0)	0.932
Total bilirubin (mg/dL)	0.8 ± 0.6	0.9 ± 0.6	0.846
AST (IU/L)^†^	35 ± 30	38 ± 46	0.759
ALT (IU/L)^†^	41 ± 30	38 ± 46	0.896
**Immunosuppressive drug dosages**			
Azathioprine (mg/day)*, (n = 2 vs 3 cases)	75	75	-
Prednisolone (mg/day), (n = 5 vs 6 cases)	7 ± 5	10 ± 12	0.567
Mycophenolate mofetil (mg/day), (n = 30 vs 27 cases)	631 ± 272	557 ± 254	0.293
Tacrolimus (mg/day), (n = 27 vs 29 cases)	2.5 ± 1.6	1.6 ± 0.9	0.009
Sirolimus (mg/day), (n = 13 vs 7 cases)	1.1 ± 0.3	1.3 ± 0.5	0.234
Cyclosporine (mg/day), (n = 9 vs 9 cases)	103 ± 49	119 ± 53	0.511
**Immunosuppressive drug levels**			
Tacrolimus (ng/mL)	4.7 ± 3.4	4.0 ± 4.2	0.536
Sirolimus (µg/L)	4.9 ± 1.5	5.9 ± 1.1	0.545
Cyclosporine (µg/L)	396 ± 174	449 ± 194	0.135

*Median.

^†^Mean ± SE.

ALT: alanine aminotransferase; AST: aspartate aminotransferase; LT: liver transplantation; ACR: acute cellular rejection.

The results of liver function tests were generally within the normal ranges, with some mild elevation of transaminase levels, and no differences between the groups. The MELD-Na score ranged from 6–40. The calculation is based on the UNOS.org allocation calculator. The median MELD-Na score at the time of enrollment in the current study was slightly elevated, at a score of 7. In addition, in some cases, the scores were as high as 10–15 (dataset). These high levels stem from the increased creatine levels due to the effect of calcineurin inhibitors and were not related to liver disease.

A comparison of immunosuppressive drug use between the two groups showed that the seropositive group used slightly higher doses of mycophenolate mofetil (631 ± 272 vs. seronegative group: 557 ± 254 mg) and tacrolimus (2.5 ± 1.6 vs. seronegative group: 1.6 ± 0.9 mg), but the difference was statistically significant only for tacrolimus (*p* = 0.009). None of the immunosuppressive drug levels were significantly different between the two groups (Table 1).

Twenty-three patients received ribavirin during the treatment of post-LT recurrent hepatitis C. Thirteen patients had received pegylated interferon/ribavirin a few years before starting the study, and 10 patients had 12-week courses of sofosbuvir/daclatasvir/ribavirin during the study period. There was no significant difference between the groups in the proportion of patients who had received ribavirin for hepatitis C (Table 1).

All four cases of ACLF were caused by a severe hepatitis B flare-up with a high HBV viral load. The explanted livers showed evidence compatible with severe hepatitis and evidence of HBsAg. Hepatitis E serology was examined in only one case, with negative results. This patient was later HEV IgG positive at the time of enrollment. The other patients were not tested for hepatitis E infection before transplantation (Supplementary Table 1).

### HEV RNA detection

Overall, 711 samples (serum 358 and feces 353) were initially tested for HEV RNA using commercial automated real-time RT-PCR and COBAS HEV (Fig. 1). The results showed 5 positive samples (serum 4 and feces 1) from 5 patients. One hundred seventeen samples showed invalid results. These included 17/358 (4.75%) serum samples and 100/353 (28.32%) feces samples. Subsequent in-house real-time RT-PCR of the 117 invalid samples showed 4 positive samples (serum 1 and feces 3) from 4 patients. When combining the results of the two methods, 7 patients had HEV RNA detected during the 12-month study period. Of these, HEV RNA was detected in the serum in 4 patients, in the feces in 2 patients, and in both the serum and the feces in 1 patient. Overall, HEV RNA was detected in 7 patients (7/91, 7.7%) in both the HEV IgG (+) and IgG (−) groups.

### HEV RNA detection in the HEV IgG (+) and IgG (−) groups

In the HEV IgG (+) group, HEV RNA was detected in the serum at the baseline in 2 patients (a, b) and in the feces at the 4th and 8th months in one patient (c), which could be considered chronic infections. Overall, RNA was detected in 3 patients in this group (3/48, 6.25%) (Table 2).

**Table 2:** The number of patients in the HEV IgG positive group. (A) and the HEV IgG negative group (B) with serum HEV serologic results and HEV RNA detection in the serum and feces at the baseline and 4, 8 and 12 months after liver transplantation.

Tests	Baseline, n/total	4 months, n/total	8 months, n/total	12 months, n/total	Total patients, n/total (%)
**A HEV IgG positive, (n = 48)**					
HEV IgG (+)	48/48	48/48	46/48	48/48	48/48 (100)
HEV IgM (+)	1/48	0/48	0/48	0/48	1/48 (2.1)
Serum RNA (+)	2/48 (a, b)	0/48	0/48	0/48	2/48 (4.2)
Feces RNA (+)	0/48	1/48 (c)	1/48 [c]	0/48	1/48 (2.1)
**B HEV IgG negative, (n = 43)**					
HEV IgG (+)	0/43	5/43	6/43	1/43	12/43 (27.9)
HEV IgM (+)	0/43	0/43	0/43	0/43	0/43 (0)
Serum RNA (+)	1/43 (d)	0/43	0/43	1/43 (e), [f]	2/43 (4.7)
Feces RNA (+)	1/43 [g]	0/43	1/43 [d]	0/43	2/43 (4.7)

The letter in parentheses (_) represents an anonymous patient who had HEV RNA detected by commercial real-time RT PCR, COBAS HEV. The letter in brackets [_] represents a patient in whom the initial result was invalid but was subsequently confirmed to be positive by in-house real-time RT PCR.

In the HEV IgG (−) group, HEV RNA was detected in the serum at the baseline in 1 patient (d) and 2 patient (e, f) at the 12th month and in the feces at the baseline in 1 patient (g) and at the 8^th^ month in 1 patient (d). In the latter case (d), HEV was detected two times, 8 months apart, which could be considered either a chronic infection or separate episodes of acute infection (Table 2). Overall, RNA was detected in 4 patients in this group (4/43, 9.3%)

### Changes in HEV serology during the follow-up period

In the HEV IgG (+) group, there was a patient who was positive for HEV IgM at the baseline (42 months after LT) without any RNA detected, but subsequent follow-up tests showed negative HEV IgM results. There were 2 patients with transient negative results for HEV IgG at the 8th month of follow-up who became positive again at the 12th month (Table 2).

In the HEV IgG (−) group, 12 patients (12/43, 27.9%) had transiently positive results for HEV IgG without any RNA detected, with 5 patients in the 4th month, 6 patients in the 8th month, and 1 patient in the 12th month (Table 2).

### Details of HEV RNA-positive cases

Seven patients had detectable HEV RNA in either their serum or feces (Table 3). Six patients were asymptomatic with normal transaminase levels. Except for one patient (f), who had a mild elevation of ALT in a subsequent follow-up study, the transaminase levels became normal without specific treatment. The cause of mild transaminitis in this patient was not confirmed; however, non-alcoholic steatohepatitis or statin-induced hepatitis was suspected. HEV RNA could not be amplified from the samples for genotyping in all patients.

**Table 3:** Characteristics of HEV RNA-positive patients during the 1-year follow-up period.

Patient	Time after LT (months)	HEV IgG	Sample site	ALT at baseline	ALT at the 4^th^ month	ALT at the 8^th^ month	ALT at the 12^th^ month	Clinical symptoms
a	20	+	s	12	13	12	11	no
b	68	+	s	17	22	44	27	no
c	61	+	f	23	19	20	24	no
d	33	−	s, f	29	31	20	31	no
e	94	−	f	23	17	44	19	no
f	128	−	s	46	72	89	37	no
g	26	−	f	10	16	11	29	no

Seven patients with laboratory details and clinical symptoms are shown. All patients had HEV RNA detected one time, except for c and d, who had HEV RNA detected twice over a 3-month period. s = serum, f = feces.

Of the seven patients in whom HEV RNA was detected, two had previous ACR requiring steroid pulse therapy, and one had post-transplant lymphoproliferative disease (PTLD) with lymphoma. The latter patient also underwent high-dose steroid therapy and chemotherapy and was subsequently cured from the disease approximately 33 months before enrollment.

### Effect of immunosuppressive drug levels on HEV RNA detection

Comparing the 7 patients with HEV RNA detected and the 84 patients without HEV RNA detected, there were no significant differences in the cyclosporine, tacrolimus and sirolimus drug levels (Table 4).

**Table 4:** Baseline immunosuppressive drug levels in the patients with HEV RNA (+) or HEV RNA (−) during the 12-month follow-up period.

Immunosuppressive drugs	HEV RNA (+) (n = 7)	HEV RNA (−) (n = 84)	P-value
Cyclosporine level (ng/mL) * (n = 1 vs 17)	555.7	414.6 ± 183.4	0.465
Tacrolimus level (ng/mL) (n = 4 vs 49)	3.2 ± 2.9	4.5 ± 3.9	0.488
Sirolimus level (ng /mL) (n = 2 vs 18)	4.9 ± 0.6	5.3 ± 1.6	0.746

Drug levels are shown as the mean ± SD, *2-hour postdose levels, n = number of cases in each group.

### Clinical course of the follow-up patients

During the 12-month follow-up period, there was no evidence of or treatment administered for significant hepatitis that was caused by hepatitis E.

## Discussion

The results of this post-LT hepatitis E study show high HEV seroprevalences (55.6%) and an active hepatitis rate of 2.8%. Further exploration in a prospective follow-up study for 12 months in 91 patients with asymptomatic post-LT cases revealed 7 patients (7.7%) with transient subclinical infection or chronic infection in both the HEV IgG (+) and HEV IgG (−) groups. As previously noted, the hepatitis E genotype 3 is the predominant genotype in Thailand, which is different from the other countries in Southeast Asia and the Indian continent^15^. The post-LT HEV seroprevalence in this study is as high as in some areas in Europe in which chronic hepatitis E and cirrhosis in patients who had undergone organ transplantation was discovered^16,17^. In the Asian region, there is only one report of a low post-LT HEV seroprevalence (2.9%) and 2 cases of viral detection with chronic hepatitis (0.12%) in Japan^13^. The HEV seroprevalence in the Thai general population varies from 10–50% depending on the area, culture and age group^6^. The HEV seroprevalence in post-LT patients in our study is quite high compared to that in the general Thai population^6^. This means that over fifty percent of members in this group had been infected either before or after their transplantation. Apart from food contamination, infection via the blood products received during the operative period might have contributed to the higher prevalence in this group^18^. However, the higher prevalence might also be partly explained by the high sensitivity of the test used in this study^19,20^. The high seroprevalence and predominant genotype 3 likely come from the consumption of undercooked pork, which is also found in other regions^21–23^.

Three patients with HEV-related hepatitis requiring therapy out of 108 post-LT recipients (2.8%) were discovered in our study. If patients with positive viral detection results were included among the asymptomatic patients (7/91 patients) during the prospective 1-year follow-up, the prevalence was 9.3% (10/108). This makes the rate of post-LT infection higher than in other reports, in which it varied from as low as 0.12% in Japan and up to approximately 3% in Europe^13,14,24^. There were two patients with chronic hepatitis E. One of the two patients, identified as being infected with HEV genotype 3, had been misdiagnosed as having steatohepatitis for a few years, and the initial HEV serology showed a negative result. There was a patient with acute hepatitis E with high transaminase levels that were detected in approximately the 8th week post-LT. This patient might have been infected by the virus via a contaminated blood product administered during the operation. A recent systematic survey in Europe and Thailand found that the prevalence of HEV RNA-contaminated blood products vary from 0.2 to 0.69 and 0.86 per 1000 donations, respectively^18,25^.

The serological test results in this study suggest that HEV IgG (+) persisted for the whole period of 12 months, but there were some fluctuations of transient negativity in the two patients, possibly due to the degree of immune suppression (Supplementary Tables 2 and 3). In the patients who were initially HEV IgG negative, 27.9% (12/43) had a transient HEV IgG positive result. The results of the HEV serological tests indicate that, even though the test is reliable for prevalence studies, it could not be used for clinical confirmation of the disease in the post-LT setting, as suggested by another study^26^.

The crucial point of this study was the prospective follow-up to explore the existence of HEV reactivation or reinfection (de novo infection), even in patients with HEV IgG (+). Our study demonstrated the existence of HEV RNA in the serum or feces of asymptomatic patients. Two of the 7 patients had positivity detected approximately 4 months apart and could be considered to have chronic infection, according to a previous recommendation^27^. In this high seroprevalence area, a figure of 7.7% (7/91 patients) for the annual incidence is a reasonable number to suggest the need for clinical vigilance. However, exploring more regarding the clinical outcomes, our data suggest silent or subclinical infections were more common than active disease during the 12-month period. The results suggest that the infection may exist and persist but that it is not necessarily a clinical issue if there no chronic hepatitis or acute hepatitis occurs during the course of clinical care.

Interestingly, a post-LT patient who had a previous HEV infection, whether the patient is HEV IgG (+) or not, still has a silent liver infection, which may emerge as a problem in the future. This is still inconclusive. Data from several animal and human studies have shown that natural HEV IgG develops after an acute HEV infection as a protective antibody against one and several genotypes. The level of protection depends on the antibody levels and wanes with time^28,29^. This partly explains the ability of the virus to cause chronic infection in immunocompromised hosts. The HEV IgG (+) in this study could be classified into 2 groups: the first was generated from a past infection before the transplantation during the immunocompetent stage, and the second was generated from a post-LT de novo infection. Several strong pieces of evidence from an animal study demonstrated the occult presence of HEV inside the livers of hosts without viremia^30^. Even so, there are very few reports in humans suggesting the possibility of the virus surviving the immune response and becoming reactivated during the immunocompromised stage^31,32^. The existence of occult HEV in patients with past HEV infection and its ability to become reactivated or to flare when the host is in an immunocompromised state remains a subject of debate.

A 12-month prospective study in Toulouse, France, in which high HEV seroprevalences in the general population (up to 50%) and in post-SOT patients (38%) were reported, explored the significant levels of HEV IgG needed for the prevention of HEV infection^33^. Twenty percent of the 263 patients were post-LT, and the rest were post-kidney transplantation. Approximately 2–3% of HEV de novo infections in the IgG (−) and reinfections in IgG (+) groups were identified in patients with evidence of hepatitis and were mainly diagnosed by serum RNA assays. The authors suggested that the low titers of HEV IgG failed to protect against de novo infection after LT rather than reactivation from occult HEV. In our 12-month prospective study, in which we found a seroprevalence of 55.6%, after excluding the patients with active disease, 3 patients (6.3%) in the IgG (+) group and 4 patients (9.3%) in the IgG (−) group had asymptomatic infections or silent infections. Nearly all patients had normal transaminase levels. No patients with active acute or chronic hepatitis were discovered. The discrepancy in the results between these two studies, especially with regard to the clinical outcome of hepatitis, might be because our study focused on only post-LT patients, who normally require less immunosuppression. As has been previously shown, not only humoral immune responses (HEV IgG) but also T-cell responses are required for the prevention of HEV infection or the development of chronic infection^34,35^. Better immune control in the post-LT setting might have resulted in only silent infections in both IgG (+) and IgG (−) patients in our study.

The technique for detecting HEV RNA in clinical samples has developed into a more standardized and automated method instead of a variety of in-house assays and a need for optimization with the World Health Organization (WHO) international standard HEV strains^36^. By the time of this study, a commercial kit for HEV detection had become available and was widely used as a standard method^37^. However, it was designed for use with serum or plasma samples. Our study yielded invalid results in approximately a quarter of the fecal samples. As the test was not developed for this purpose and there is a possibility of some inhibitors of the reactions, we decided to use an in-house real-time RT-PCR to confirm the presence of HEV RNA. The test was previously standardized with the WHO’s standard samples and used for the assessment of HEV RNA in blood donors^18^. Using this method, two more patients were identified, which yielded a total of 7 patients (7/91, 7.7%) in the final results. In another two patients with initially invalid results, HEV RNA was detected by both methods at different time points. These reaffirmed the accuracy of the in-house test. The HEV RNA was amplified and characterized as HEV genotype 3 (Supplementary Fig. 1) in one of the two patients with chronic hepatitis E, who was categorized as having active disease a few months before the beginning of the study. However, the samples from the seven patients discovered to be positive during this cohort study did not yield amplified RNA. This could be due to the very low level of RNA in silent infections.

There are some other limitations in this study. Twenty-five percent of the patients had a history of hepatitis C treatment with either pegylated interferon/ribavirin or direct-acting antivirals (DAAs) plus ribavirin. Of these, 10 patients underwent a 12-week course of sofosbuvir/daclatasvir/ribavirin during the study period. A prolonged course of pegylated interferon or ribavirin could suppress or eliminate HEV infection^38,39^. In addition, recent data indicate that sofosbuvir can suppress and has a synergistic effect on HEV viral replication^40^. These medications might have inevitably affected the number of positive cases in this study. Last, because the HEV serologies of all patients were not assessed during the pretransplant period, the positive results for HEV IgG could not differentiate between the pre- and posttransplant acquisition of HEV infections.

In summary, HEV genotype 3, which is known to cause chronic liver disease in immunocompromised hosts, is the major genotype among infected individuals in Thailand. Our study shows a very high HEV seroprevalence in post-LT cases. A further 12-month follow-up study in asymptomatic patients with mostly normal liver enzyme levels demonstrated a significant number of patients with silent infections. The detection of HEV RNA in the serum or feces of the patients did not depend on the HEV IgG seropositivity. More importantly, these occult or subclinical infections did not become overt infections or clinically significant hepatitis.

## Methods

Post-LT patients receiving care at the King Chulalongkorn Memorial Hospital were approached for study enrollment between October 2015 and June 2016. The study protocol is shown in Fig. 1. A total of 108 patients provided a 10-mL blood sample (for serum testing for HEV IgG and IgM antibodies, and HEV RNA detection) and a fresh feces sample (for HEV RNA detection). Each patient’s demographic and clinical data, including the patient’s medical history; current medication(s), especially immunosuppressive agents; and laboratory results were retrieved from the medical records. Patients who had a previous relevant history, active acute or chronic hepatitis E, or active malignancy and those who were unable to attend regular follow-up visits were excluded from the study. After recruitment, the patients attended follow-up visits every 4 months for 12 months for a clinical assessment, basic laboratory tests, and blood/feces sample collection. All samples were stored at −70 °C and simultaneously tested at the end of the study.

This study was approved by the Chulalongkorn University Institutional Review Board, IRB number 028/59. Written (signature or thumbprint) informed consent was obtained from all patients prior to study participation. The study was registered with the Thai Clinical Trials Registry, TCTR20170112005, on 12 January 2017. During the 12-month follow-up period, all participants who had active hepatitis were investigated and treated according to standard medical care, including for hepatitis E-related diseases. After the final test results of the study were obtained, the patients were informed of their status and counseled according to the Declaration of Helsinki and Ethical Guidelines for Clinical Research.

### Laboratory methods

#### HEV serology assays

All serum samples were tested for the IgG and IgM HEV antibodies using an anti-HEV 96-well plate enzyme-linked immunosorbent assay (ELISA) (WE-7296, WE-7196; WANTAI Biological Pharmacy Enterprise, Beijing, China) according to the manufacturer’s instructions. The optical density/cut-off (OD/co) values above 1.1 were considered positive. Values between 0.9 and 1.1 were considered negative.

#### HEV RNA detection

The levels of HEV RNA in the serum and feces samples were evaluated using the COBAS HEV Test, which is a fully automated molecular diagnostic system for the hepatitis E virus. The assay was performed on the COBAS 6800 System, Roche Molecular Diagnostics, Rotkreuz, Switzerland, according to the manufacturer’s instructions. The 95% limit of detection (LoD) was 18.6 IU/ml. If the test showed invalid results, an in-house real-time RT-PCR was used for confirmation.

For the in-house real-time RT-PCR, the HEV RNA was detected by one-step real-time PCR for HEV ORF2/3^41^. The 95% LoD of HEV RNA detection, as calibrated against the WHO international standard sample, was 53.5 IU/mL^18^.

#### HEV genotyping

The PCR product from a semi-nested RT-PCR was used to determine the genotype of HEV present in samples with the published primers for ORF1 and ORF2^42^. The amplicons were sequenced and analyzed with Chromas LITE (v2.1.1), BioEdit v.7.0.4.1 and subjected to a BLASTN search (http://www.ncbi.nlm.nih.gov). Phylogenetic trees were generated from the partial nucleotide sequences of the ORF1 (315 base pairs) and ORF2 (417 base pairs) regions (Supplementary Fig. 1). The neighbor-joining tree method was implemented in MEGA v.6.0. The reliability of the phylogenetic trees was assessed by 1000 bootstrap resampling.

#### Statistical analysis

All data were collected in MICROSOFT EXCEL 2010 (www.microsoft.com) and analyzed using IBM SPSS Statistics version 23 for Windows, NY: IBM Corp, USA. The continuous variables were compared using Student’s t test, and a p-value <0.05 was considered significant.

## Supplementary information

Supplementary information.

Supplementary information2.

## Data Availability

The dataset of this work is submitted in Dryad (ID: 10.5061/dryad.5dv41ns38 or using this temporary link: https://datadryad.org/stash/share/a_OjGpiTYPvzY5XNFS7tNgi-tyYsS3CpY9y07W_Qh38).
