# Supplementary material for: Clinical significance of post-liver transplant hepatitis E seropositivity in high prevalence area of hepatitis E genotype 3: a prospective study
Source: Sci Rep. 2020 Apr 30;10:7352. doi: 10.1038/s41598-020-64551-x (PMC7192897; doi:10.1038/s41598-020-64551-x)
Supplement: Supplementary file 1 — Supplementary information. [file 41598_2020_64551_MOESM1_ESM.pdf]

Supplementary information

Clinical significance of post-liver transplant hepatitis E seropositivity in high prevalence area of hepatitis E genotype 3: a prospective study

Piyawat Komolmit*[1, 2], Vinita Oranrap[1], Sirinporn Suksawatamnuay[2], Kessarin Thanapirom[1, 2], Supachaya Sriphoosanaphan[1, 2], Nunthiya Srisoonthorn[1, 2], Nawarat Posuwan[3], Thanunrat Thongmee[3], Sombat Treeprasertsuk[1], and Yong Poovorawan[3]

1. Division of Gastroenterology, Department of Medicine, Faculty of Medicine, Chulalongkorn University, Bangkok, Thailand
2. Center of Excellence in Liver Diseases, King Chulalongkorn Memorial Hospital, Bangkok, Thailand
3. Center of Excellence in Clinical Virology, Faculty of Medicine, Chulalongkorn University, Bangkok, Thailand

*Corresponding Author: Piyawat Komolmit, M.D., Ph.D
Faculty of Medicine, Department of Medicine, Division of Gastroenterology, Chulalongkorn University, King Chulalongkorn Memorial Hospital, Rama 4 Road, Pathumwan, Bangkok 10330, Thailand
E-mail: pkomolmit@yahoo.co.uk
Tel: +66-947825195

Table of contents

1. Supplementary Figure 1. Phylogenetic tree based on the partial HEV ORF1 (**A**) and ORF2 (**B**) sequences derived from the serum sample of one of the two patients with chronic hepatitis E.

2. Supplementary Table 1. Four enrolled cases who had ACLF as an indication for LT

3. Response to a reviewer comment regarding the episodes of negative HEV IgG in two patients and supplementary table 2 and supplementary table 3

Supplementary table 2 HEV IgG titer of patient BK49 in the study cohort

Supplementary table 3 HEV IgG titer and cyclosporin level of patient BK42

Supplementary Figure 1. Phylogenetic tree based on the partial HEV ORF1 (**A**) and ORF2 (**B**) sequences derived from the serum sample of one of the two patients with chronic hepatitis E.

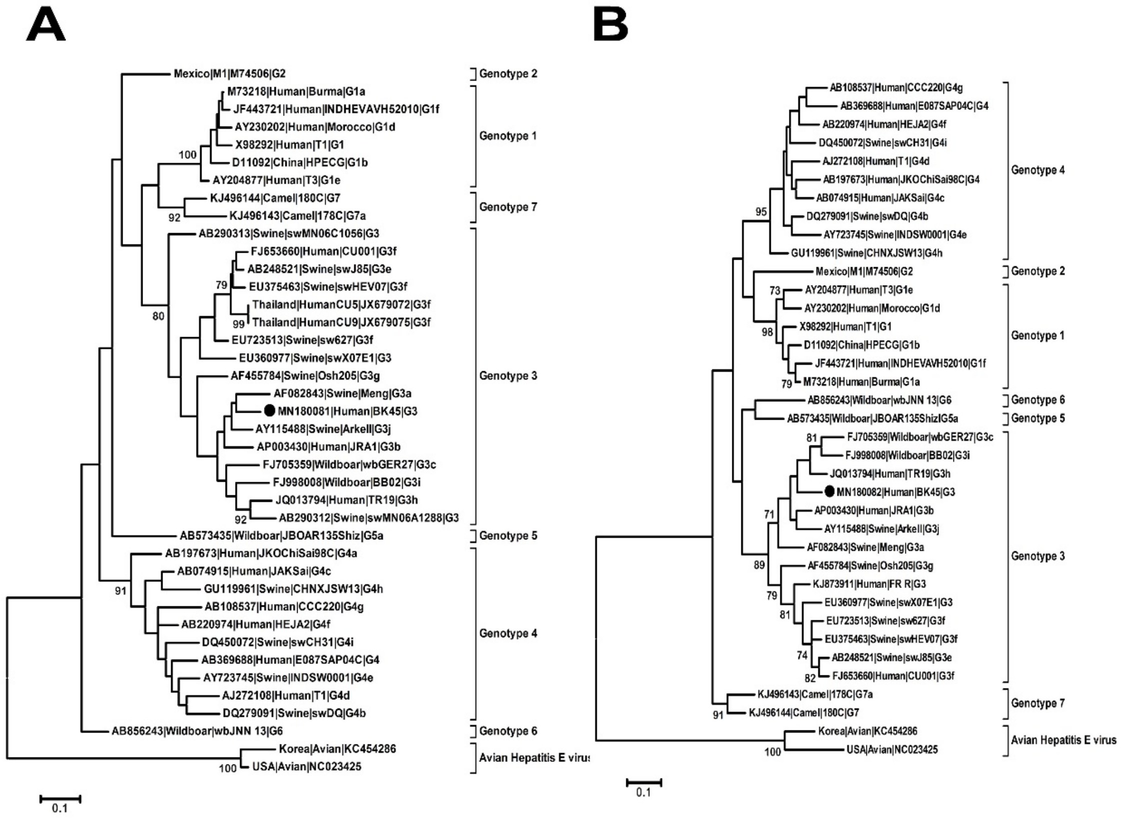

Note: The nucleotide sequences were compared with the reference strains using the neighbor-joining method with bootstrap consensus inferred from 1000 replicates. Bootstrap values > 70% are shown at the branch nodes. Scale bar represents a nucleotide substitution rate. (GenBank database accession: MN180081 and MN180082)

Supplementary table 1. Four enrolled patients who had ACLF as an indication for LT

Case	LT to enrollment	Cause	HEV serology pre-LT	HEV IgG at the enrollment
1	1 year, 5 months	hepatitis B flare up	HEV IgG negative<br>HEV IgM negative	positive
2	7 year, 10 months	hepatitis B flare up	not assessed	positive
3	8 years, 8 months	hepatitis B flare up	not assessed	positive
4	9 years, 6 months	hepatitis B flare up	not assessed	negative

ACLF: acute on chronic liver failure; LT: liver transplantation

Response to a reviewer comment regarding the episodes of negative HEV IgG in two patients at the 8th month

Comment: Some of the laboratory data are unusual and needed further evaluation. For example, one person was positive for IgG anti-HEV at 0, 4 and 12 months, but was negative at 8 mo. How do the authors explain this? What were the Sample/cut-off OD ratios at each time point? It would have been useful to run these specimens again – all in the same assay.

Answer: We hypothesize that the overimmunosuppressive status of the patients might result in a lower antibody titer and cause an episode of negative results at the 8th month. The HEV IgG titers of the first patient, BK49, are shown in supplementary table 2. He had a history of hepatic post-transplant lymphoproliferative disease (PTLD) and lymphoma during the first year post-LT. He had curative treatment with high-dose steroid therapy and chemotherapy. He was enrolled in this study at approximately the 5th year after LT. We hypothesize that this patient had a long-term low immunosuppressive status related to his underlying condition. This could be reflected by the low HEV IgG titers, the value of which was just above one most of the year and less than one at the 8th month.

Supplementary table 2 HEV IgG titer of patient BK49 in the study cohort

Case	Serum HEV IgG assay at							
	Baseline		4th month		8th month		12th month	
	Titer*	Result	Titer*	Result	Titer*	Result	Titer*	Result
BK49	1.20	+	1.15	+	0.21	-	1.51	+

Titer: A/C.O. (A: Absorbance value, C.O.: Cut-off value)
A/C.O. <1 = negative (-)
A/C.O. 0.9-1.1 = borderline
A/C.O. >1.1 = positive (+)

The second patient, BK42, had quite high HEV IgG titers all year, except for the negative result at the 8th month (supplementary table 3). We examined the details of the immunosuppressive levels of the patient and found fluctuations in the cyclosporin level that could probably explain the negative result. The usual cyclosporin level of this patient over several years was approximately 400-500 ng/mL. At the 4th month, for an unknown reason, the level was low, at 127 ng/mL, which could result in his higher immune response and strong positive HEV IgG titer. By the 8th month, his cyclosporin level had increased 6.5 times. We postulate that his immune status was low due to the high cyclosporin level, resulting in a decrease in the HEV IgG titer to less than one. By the 12th month, his immune status had successfully adapted, resulting in an increased immune status and an increase in the HEV IgG titer to 14.82.

Supplementary table 3 HEV IgG titer and cyclosporin level of patient BK42

Case and drug levels	Serum HEV IgG assay at							
	Baseline		4th month		8th month		12th month	
	Titer*	Result	Titer*	Result	Titer*	Result	Titer*	Result
BK42	>16.58	+	>16.58	+	0.20	-	14.82	+
Cyclosporin levels†	520		127		824		1000.8	

* Titer: A/C.O. (A: Absorbance value, C.O.: Cut-off value)
† 2-hour post dose in ng/mL

Overall, we hypothesize that fluctuations in the immune status of the patients influence the immune response in terms of the HEV IgG titer.

Of note, all samples were tested for HEV antibodies using the WANTAI test at the same time at the end of the study.
