# Supplementary figures and images for: Clinical significance of post-liver transplant hepatitis E seropositivity in high prevalence area of hepatitis E genotype 3: a prospective study
Source: Sci Rep. 2020 Apr 30;10:7352. doi: 10.1038/s41598-020-64551-x (PMC7192897; doi:10.1038/s41598-020-64551-x)

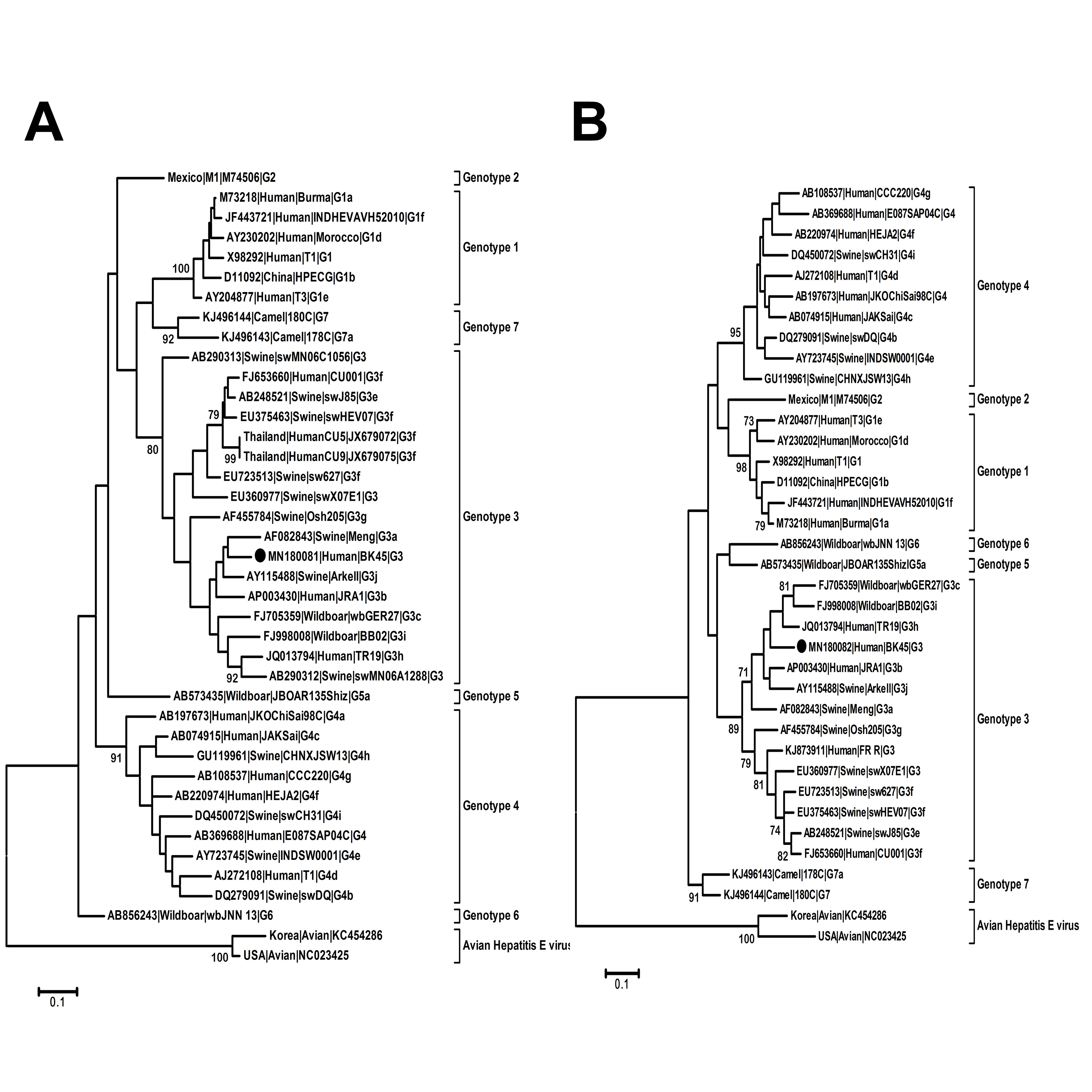

Supplement: Supplementary file 2 — Supplementary information2. [file 41598_2020_64551_MOESM2_ESM.jpg]
